# Insertional protein engineering for analytical molecular sensing

**DOI:** 10.1186/1475-2859-5-15

**Published:** 2006-04-03

**Authors:** Rosa María Ferraz, Andrea Vera, Anna Arís, Antonio Villaverde

**Affiliations:** 1 Institut de Biotecnologia i de Biomedicina and Departament de Genètica i de Microbiologia, Universitat Autònoma de Barcelona, Bellaterra, 08193 Barcelona, Spain; 2 Departament de Matemática Aplicada IV, Universitat Politècnica de Catalunya, Campus Nord, Jordi Girona, 1-3, 08034 Barcelona, Spain

## Abstract

The quantitative detection of low analyte concentrations in complex samples is becoming an urgent need in biomedical, food and environmental fields. Biosensors, being hybrid devices composed by a biological receptor and a signal transducer, represent valuable alternatives to non biological analytical instruments because of the high specificity of the biomolecular recognition. The vast range of existing protein ligands enable those macromolecules to be used as efficient receptors to cover a diversity of applications. In addition, appropriate protein engineering approaches enable further improvement of the receptor functioning such as enhancing affinity or specificity in the ligand binding. Recently, several protein-only sensors are being developed, in which either both the receptor and signal transducer are parts of the same protein, or that use the whole cell where the protein is produced as transducer. In both cases, as no further chemical coupling is required, the production process is very convenient. However, protein platforms, being rather rigid, restrict the proper signal transduction that necessarily occurs through ligand-induced conformational changes. In this context, insertional protein engineering offers the possibility to develop new devices, efficiently responding to ligand interaction by dramatic conformational changes, in which the specificity and magnitude of the sensing response can be adjusted up to a convenient level for specific analyte species. In this report we will discuss the major engineering approaches taken for the designing of such instruments as well as the relevant examples of resulting protein-only biosensors.

## Review

### Introduction

Conventional biosensors are hybrid elements consisting of a biochemical receptor for a given analyte, physically coupled to a physicochemical transducer that converts such interaction into a macroscopic, analytically useful signal [1]. In the last decades, many types of biosensors have been under continuous development, integrating biological components such as proteins, nucleic acids, membranes cells and even tissues acting as receptors, and different signal transducers devices including microbalances, electrodes, optical components and semiconductors. Such instruments have been applied into a diversity of fields but specially for the detection of contaminants in foods and environment [2]. More recently, and pressured by the need of more sensitive and specific detection tools for biomedical applications, in particular diagnosis, new types of protein-only biosensors are being explored [3], that contain both the receptor and transducer elements in a single polypeptide chain. Alternatively, protein-only sensors can specifically act as receptors that exploit the whole living cell where they are synthesised, as a complex signal transducer, enabling the detection of the analyte through intricate global activities such as differential growth or support of viral multiplication among others. Both type of protein-only biosensors offer very appealing advantages over classical devices. First, chemical coupling to the signal transducer is not required as straightforward bioproduction results in a ready-to-use final product, either a purified protein of protein-producing cells or cell extracts. Also, protein engineering procedures such as site-directed mutagenesis or directed molecular evolution allow refining the specificity of ligand binding and permit the development of new receptors for new analytes, as demanded from medicine or industry.

**Figure 1:**
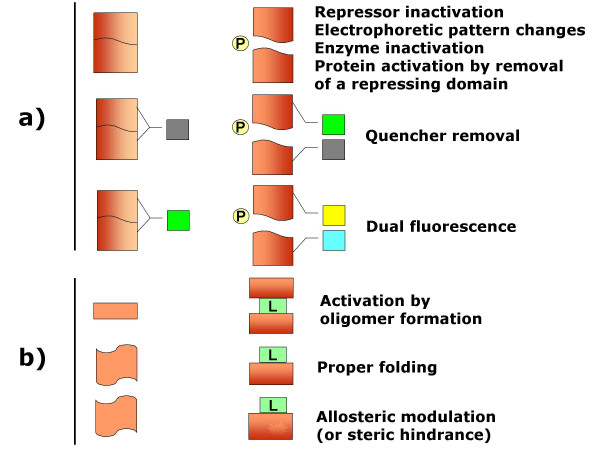
The biosensing principles of the constructs listed in Table 1 are summarized here as split in two groups. In **a)**, the sensing principles underlying cleavable platforms are presented in which simple hydrolysis of protease target site-bearing hybrid proteins by an effector protease (P) result in a macroscopic signal. Among others, variations of the migration pattern, enzyme activation or inactivation, repressor inactivation, enhanced fluorescence by removal of a quencher or dual fluorescence emission by FRET modulation. In **b)**, a ligand (L) promotes conformational modifications in the sensor either multimerization, correct folding or allosteric activation. A few enzyme biosensors are inactivated in presence of the ligand probably by steric hindrance of the active site.

The mechanics for which a protein responds to a specific ligand in a macroscopically detectable way would generally imply variations in its activity, either enhancement or inhibition, that could be detectable either directly or indirectly through a biological amplification process. In general, natural protein-ligand interactions result in moderate conformational modifications that would be poorly useful in molecular switching, as they have a limited impact on protein's activity. Protein engineering allows the modification of the receptor in a way in which the interaction with the analyte promotes profound conformational modifications. There are many examples of useful intracellular indicators of molecular interactions, gene expression or for biological screening [4] that use at different extent end-to-end fusion proteins, including two-hybrid systems [5], fluorescence resonance energy transfer (FRET) [6], and protein fragment complementation [7] among others. However, insertional protein engineering allows a more versatile combination of functional modules for the construction of highly responsive mosaic proteins exhibiting unusual conformational versatility upon ligand binding [8,9]. Obviously, the protein segment or domain acting as a receptor element must be conveniently displayed on the protein surface to allow a proper interaction with the analyte. Although some of the constructs referenced below derive from random insertions and further selection [10,11], the previous identification of solvent-exposed permissive sites through different procedures has allowed a more rational designing procedures based on site directed peptide insertion for the construction of biosensors and other type of multifunctional proteins [12-19]. The principles of protein functionality supporting insertional approaches for biosensor construction are further discussed as exemplified by representative models and specific applications, being most of the resulting protein-only biosensors based on either cleavable (Figure 1A) or allosteric (Figure 1B) protein platforms. Representative examples of specific sensors and construction approaches are listed in Table 1.

**Table 1:** Representative examples of protein only biosensors obtained by insertional mutagenesis.

Holding protein	Strategy	Insert	Analyte	Sensing mechanism	Signal (factor, when activated)	Application (proved or suggested)	References
β-galactosidase	Site directed insertion	FMDV^a ^and HIV antigenic peptides	Anti-peptide antibodies and immune sera	Allosteric	Enzymatic activity up-shift (up to 12-fold)	Diagnosis	[38,39,43,47,48,49,59]
β-galactosidase	Site directed insertion	HIV protease substrate	HIV protease	Cleavage mediated inactivation	Enzymatic activity down-shift or electrophoretic analysis	Antiviral drug design and screening	[25,26]
Alkaline phosphatase	Site directed insertion	HIV antigenic peptide	Anti-peptide antibodies	Probably steric hindrance	Enzymatic activity down-shift	Diagnosis	[46]
Alkaline phosphatase	Site directed insertion plus site directed mutagenesis of the active site	HIV and HCV antigenic peptide	Anti-peptide antibodies	Allosteric	Enzymatic activity up-shift (up to 2.5-fold)	Diagnosis	[40]
GFP	Site directed insertion followed by random mutagenesis	TEM1 β-lactamase	TEM1 β-lactamase inhibitor	Allosteric	Fluorescence emission up-shift (not determined)	Drug design and screening	[41,42]
EGFP	Amino acid replacement	LPS/LA-binding motif	Bacterial LPS	Quenching	Fluorescence emission down-shift	Quality control (endotoxin detection)	[60]
TEM β-lactamase	Random insertion and phage-mediated selection	Random peptides	Anti PSA antibodies	Allosteric and steric hindrance upon the specific construct	Enzymatic activity down- or up-shift (up to 1.7-fold)	Diagnosis	[10]
p53	Site directed insertion plus site directed deletion	LF, HA and HSV antigenic peptides	Anti-peptide antibodies	Dimerization	Electrophoretic mobility up-shift (up to 100-fold)	Diagnosis and screening	[28]
p53	Site directed insertion	HIV and LF protease substrates	HIV protease and LF	Auto-inhibitory domain removal	Electrophoretic mobility up-shift (up to > 100-fold) or in situ hybridisation (2-fold)	Screening	[28]
cI lambda repressor	Site directed insertion	HIV, HCV and SARS protease substrates	HIV, HCV and SARS proteases	Cleavage mediated inactivation	Phage plaques counting (up to 50-fold)	Antiviral drug design and screening	[32,33,61]
MBP	Site directed insertion eventually followed by punctual mutagenesis	Zinc binding sites	Zinc	Allosteric	Fluorescence emission modulation (up to 8-fold)	Not specified, presumably wide	[62]
MBP	Random insertion	TEM-1 beta-lactamase segment	Maltose and other sugars	Allosteric	Enzymatic activity up-shift (up to 1.7-fold)	Not specified, presumably wide	[11]
DHFR	Site directed insertion eventually followed by punctual mutagenesis	FKBP macrolide- binding protein and ERα ligand binding domain	FK506 and estrogen	Binding-promoted thermostability and consequent genetic complementation	Growth of temperature-sensitive yeast under non-permissive temperatures (up to 2.5-fold)	Drug design and screening	[56]
FynSH3 ^b^	Deletion	none	Proline-rich peptide ligand	Ligand induced protein folding	Tryptophan fluorescence increase (up to 15-fold)	Not specified, presumably wide	[55]
GFP-DsRed fusion ^b^	Modular fusion	TEV protease substrate	TEV protease	Cleavage mediated fluorescent tag separation	Dual fluorescent emission yield	Antiviral drug design and screening	[29]

^a ^Abbreviations are explained in the abbreviation list.

^b ^A few examples of protein sensors obtained by either deletion or end-to-end fusion approaches are also shown.

### Cleavable platforms

The most dramatic conformational modification that a given ligand (in this case a protease) might induce on a target protein is hydrolysis, that mostly result in its functional inactivation but being sometimes a requisite for a polypeptide reaching the active form, if existing as an inactive precursor. In fact, targeted proteolysis is a biological principle regulating many complex cellular events [20-22]. Therefore, including a specific protease target site on a protein's surface would made it susceptible to site-limited digestion resulting in detectable changes in its electrophoretic pattern. The successful implementation of such technology would imply a refined analysis of the protease target site susceptibility, as peptide display in different solvent-exposed sites could result in distinguishable digestion efficiencies, since the protein regions neighbouring the insert seem to have a dramatic influence on the peptide conformation [23]. This has been exemplified by the insertional mutagenesis of the protease resistant, green fluorescent protein (GFP), to make it susceptible to trypsin and other proteolytic enzymes [24]. The detection of specific proteases and proteolytic activities is now of extreme relevance in virology and in particular for designing antiviral drugs that inhibit viral protein processing and therefore multiplication. Beyond the straightforward electrophoretic analysis of the sensing protein [25], a rather inconvenient technique from the analytical point of view, monitoring protease-mediated reduction of activity (fluorescence emission or enzymatic activity) would offer a more convenient protease sensing signal [26]. In a step further, it is known that many natural proteins are proteolytically activated by the removal of self inhibitory protein domains [27]. In this context, the convenient insertional engineering of the carboxy terminus of p53, containing such a regulatory element, has resulted in a set of p53 variants that are activated upon its removal mediated by either the lethal factor (LF) or the human immunodeficiency virus (HIV) protease [28]. Again, in this case, the sensing signal is detectable by up-shift electrophoretic analysis, since the activated p53 gains the ability to interact with specific DNA sequences [28].

In an attempt to produce more convenient analytical signals, protease target sites have been introduced in the linker between two end-to-end fused proteins that emit fluorescence at different wavelengths, so the cleavage can be monitored by variations in the FRET spectra [29-31]. Although being not a standard insertional approach, the principles governing such engineering processes are similar to those discussed above. In this context, the protein hydrolysis splitting a fluorophore and its quencher has been also a successfully proven biosensing principle [60].

It would be expected that the generation of a signal by a specific proteolytic attack acted as an all-or-nothing switcher rather than as a fine sensing tool. However, a very discriminative monitoring tool for viral proteases activity was implemented as a high-throughput analytical method for antiviral drug testing and evaluation of the enzyme activity. The cI lytic repressor of the *E. coli *lambda bacteriophage has been engineered to accommodate a selected target site for proteases from either HIV [32], hepatitis C virus (HCV) [33] and severe acute respiratory syndrome (SARS) viruses [34]. The appropriate co-expression of the engineered cI and the protease promotes lytic lambda propagation that is reported by plaque counting. This system serves not only to test protease inhibitors for antiviral drug research but also to quantitatively evaluate the activity of proteases from mutant viruses emerging in patients treated with antiviral, protease-targeted drugs [35]. The cascade events supported by the cell as a network signal transducer permits the quantitative translation of the statistic cI hydrolysis within the cellular pool, what would be probably not possible by using a more straightforward signal transducing system.

### Allosteric platforms

The regulatable activity of allosteric enzymes lies on a biological principle highly matching with the protein-only biosensing concept [36]. The activity of allosteric enzymes is modulated upon binding of an effector to a receptor site, that being different from the active site, can influence its performance through the conformational impact promoted by the allosteric effector. Since most natural effectors are irrelevant for analytical purposes, both allosteric and non allosteric enzymes have been engineered to allosterically respond to new effectors by insertion of appropriate receptor sites, in some cases accompanied by directed or random mutagenesis of the enzyme or directed molecular evolution. This straightforward insertional strategy often requires the identification of permissive sites in which inserted motives do not disturb irreversibly the enzyme activity [12,37], and has proven to be efficient in the engineering of β-galactosidase [38,39], alkaline phosphatase [40], β-lactamase [10] and GFP [41,42] as allosteric biosensors. As the fine mechanics of the conformational signal transduction in allosteric activation is not know, such devices have been constructed by error-and-trial approaches. Recently [11], a random insertional approach has permitted to newly create two allosteric enzymes by domain incorporation, by a strategy, in principle, with general applicability in biosensor design. Among enzyme inhibitors and other few ligand species that activate allosteric biosensors, antibodies have been noted to be specially efficient allosteric effectors [36] and the use of antigenic peptides as receptors in only-protein biosensors would offer appealing tools for the fast molecular diagnosis of infectious diseases [39,43]. Allosteric β-galactosidases displaying arginine-glycine-aspartic acid (RGD)-containing antigenic peptides [23], are activated by anti-peptide antibodies [38] but not by RGD-targeted integrins binding the same receptor [44]. This fact indicates different conformational constraints in the binding of both molecules [45] and suggests that the adaptive antibody binding could be a major force in sensor activation.

A main problem of allosteric biosensing is the poor signal-background ratio, that in most of cases does not reach more than 2-fold (Table 1). Higher activation factors would be extremely desirable for fine analytical applications where a wide dynamic range is required. In fact, in a few examples, the presence of anti-peptide antibodies (the analyte) even reduces the activity of the enzyme probably by steric hindrance of the active site, as reported by alkaline phosphatase [46] or β-lactamase [10], when the antigenic peptide was placed in the very close vicinity of the active site. The inhibition of the enzymatic activity is not very desirable as an analytical signal since in high-throughput analysis of complex samples, the presence of enzyme inhibitors might render false positives. By comparing different species of allosteric biosensors sensing anti-peptide antibodies, it was recognized that the activation factor was highly depending on the perturbation that the inserted peptide receptor had promoted on the activity of the enzyme platform after insertion [47]. Greater was the reduction in the specific activity of the enzyme, higher the activation mediated by the effector, but reaching only activation factors around 2 that seemed to be a biological upper limit to allosteric activation [47]. However, a deeper exploration of β-galactosidase allosteric sensors has revealed that the signal background ratio can be enhanced up to more than 10-fold, by alternative or combined approaches such as introducing a higher number of receptors per enzyme [43,48], optimising the reaction conditions [49] and selecting the appropriate substrate [50].

Apart from plain diagnostic utilities, allosteric sensors can intriguingly perform as tools for the analysis of the immune response, as they specially recognize antibodies with a high potential as modifiers of the epitope conformation. In this context, a β-galactosidase sensor displaying an HIV gp41 epitope and responsive to human HIV-immune sera is preferentially activated by the IgG4 antibody subpopulation [51]. As at least in the case of HIV infection the ability of anti-viral antibodies to modify the epitope's conformation is strongly related to their neutralizing activity [52,53] and probably to the progression of the infection [54], allosteric biosensing could eventually offer a valuable instrument for high-throughput sera analysis for prognostic investigation.

### Other examples of conformation-dependent sensor activation

Conformational changes promoted by molecular interactions may generate signals suitable for biosensing other than allosteric responses. The correct folding of a deletion mutant of the human Fyn tyrosine kinase (FynSH3), a predominantly β-sheet protein, is induced by the binding of an appropriate proline-rich peptide ligand, and the folding process monitored in real time by tryptophan fluorescence [55]. Temperature-sensitive yeast cells lacking dihydropholate reductase (DHFR) are complemented by two mouse DHFR containing foreign different ligand binding domains [56]. Culture growth is then enhanced in presence of the respective ligands proving that molecular binding activates the complementing enzyme. Although this system can be observed as a generic biosensor [57], its real potential would lie on the selection of specific or improved ligands by directed molecular-cellular evolution. On the other hand, the presence of bivalent antibodies can promote dimer formation of a mutant p53 in which the tetramerization domain has been removed and antigenic B-cell epitopes of viral origin conveniently inserted [28]. Since dimers are much more active than monomers, the presence of antiviral antibodies enables p53 to bind DNA in an electrophoretically detectable manner.

Other conformation-linked effects of molecular interactions might also result in detectable activity changes or phenotypes acting as macroscopic signals for a given analyte. Gaining further knowledge about enzyme structure and dynamics would necessarily offer additional possibilities of rational protein engineering [58] for exploitation of such conformational signals.

## Conclusion

Insertion of foreign peptides as receptors of protein-only biosensors confers the resulting protein construct the ability to sense analytes by dramatic conformational changes unusual in the native, non engineered protein. For such a sensor being efficiently responsive, appropriated permissive sites need to be selected permitting proper receptor display and signal transduction, and the whole protein might require further engineering to gain specificity and response range. Although most protein-only biosensors derive from trial-and-error engineering approaches, rational and very clever setting-ups are exemplified by combinations of sensing protein segments and conveniently modified acceptor proteins. Among the diversity of sensing strategies based on insertional mutagenesis two protein platforms emerge as the most explored, namely cleavable sensors responding to proteases or their inhibitors, and allosteric, among whose most efficient effectors are antibodies. The performance of these two sensor types has been largely proved in the high throughput screening of antiviral drugs and for the molecular diagnosis of infectious diseases respectively. Although the potential applications of protein-only biosensors are diverse and still have to be fully exploited, they have arisen as valuable new tools in biomedicine being intriguing alternatives to classical sensing technologies.

## Authors' contributions

Rosa M Ferraz and Andrea Vera have equally contributed to this review.

## Abbreviations

DHFR Dihydropholate reductase

DsRed Engineered mutant of red fluorescent protein

EGFP Enhanced green fluorescent protein

FMDV Food-and-mouth disease virus

FRET Fluorescence resonance energy transfer

GFP Green fluorescent protein

HA Influenza hemaglutinin

HCV Hepatitis C virus

HIV Human immunodeficiency virus

HSV Herpes simplex virus

LA Lipid A

LF Lethal factor

LPS Lipopolysaccharide

MBP Maltose binding protein

RGD Arginine-glycine-aspartic acid tri-peptide

SARS Severe acute respiratory syndrome

TEV Tobacco etch virus

TEM β lactamase

PSA Prostate specific antigen
